# The emerging role and therapeutical implications of ferroptosis in wound healing

**DOI:** 10.1093/burnst/tkae082

**Published:** 2025-02-14

**Authors:** Yanan Zhao, Zhiyang Chen, Shenghao Xie, Feng Xiao, Qian Hu, Zhenyu Ju

**Affiliations:** Key Laboratory of Regenerative Medicine of Ministry of Education, Institute of Aging and Regenerative Medicine, Department of Developmental & Regenerative Medicine, College of Life Science and Technology, Jinan University, No. 601, Huangpu Avenue West, Tianhe District, Guangzhou, 510632, China; Key Laboratory of Regenerative Medicine of Ministry of Education, Institute of Aging and Regenerative Medicine, Department of Developmental & Regenerative Medicine, College of Life Science and Technology, Jinan University, No. 601, Huangpu Avenue West, Tianhe District, Guangzhou, 510632, China; Key Laboratory of Regenerative Medicine of Ministry of Education, Institute of Aging and Regenerative Medicine, Department of Developmental & Regenerative Medicine, College of Life Science and Technology, Jinan University, No. 601, Huangpu Avenue West, Tianhe District, Guangzhou, 510632, China; Key Laboratory of Regenerative Medicine of Ministry of Education, Institute of Aging and Regenerative Medicine, Department of Developmental & Regenerative Medicine, College of Life Science and Technology, Jinan University, No. 601, Huangpu Avenue West, Tianhe District, Guangzhou, 510632, China; Key Laboratory of Regenerative Medicine of Ministry of Education, Institute of Aging and Regenerative Medicine, Department of Developmental & Regenerative Medicine, College of Life Science and Technology, Jinan University, No. 601, Huangpu Avenue West, Tianhe District, Guangzhou, 510632, China; Key Laboratory of Regenerative Medicine of Ministry of Education, Institute of Aging and Regenerative Medicine, Department of Developmental & Regenerative Medicine, College of Life Science and Technology, Jinan University, No. 601, Huangpu Avenue West, Tianhe District, Guangzhou, 510632, China

**Keywords:** Wound healing, Ferroptosis, Iron, Lipid peroxidation, Therapies

## Abstract

Wound healing is a complex biological process involving multiple steps, including hemostasis, inflammation, proliferation, and remodeling. A novel form of regulated cell death, ferroptosis, has garnered attention because of its involvement in these processes. Ferroptosis is characterized by the accumulation of lipid peroxides and is tightly regulated by lipid metabolism, iron metabolism, and the lipid-peroxide repair network, all of which exert a significant influence on wound healing. This review highlights the current findings and emerging concepts regarding the multifaceted roles of ferroptosis throughout the stages of normal and chronic wound healing. Additionally, the potential of targeted interventions aimed at modulating ferroptosis to improve wound-healing outcomes is discussed.

Highlights

- Wound healing is a complex biological process that involves multiple steps.
- Ferroptosis is an emerging form of regulated cell death caused by iron-dependent lipid peroxidation. It is regulated by lipid metabolism, iron metabolism, and lipid peroxide repair network.
- Ferroptosis is involved in different stages of normal wound healing and chronic wound healing.
- Ferroptosis holds promise as a potential target for enhancing wound healing.

## Background

Wounds are widely acknowledged as prevalent and persistent clinical conditions that impose substantial economic costs and social challenges on patients. Wound healing is a complex and dynamic process that is essential for recovering the structural and functional integrity of tissues. This process involves a well-coordinated series of biological events including hemostasis, inflammation, proliferation, and remodeling [1]. Understanding these mechanisms not only addresses fundamental biological questions but also paves the way for innovative treatments and interventions that can accelerate and optimize tissue repair in diverse clinical scenarios. Ferroptosis, a nonapoptotic form of cell death driven by iron-dependent lipid peroxidation, has garnered significant attention in recent years because of its implications in various pathological conditions, including ischemia–reperfusion injury (IRI), neurodegenerative diseases, acute heart injury, and cancer [2]. Ferroptosis inhibitors represent a promising class of compounds with the potential to address these conditions. Their therapeutic applications could extend beyond neurodegeneration and cardiovascular diseases to cancer and other diseases in which ferroptosis plays a significant role. Intriguingly, current research underscores the critical role of ferroptosis throughout the wound-healing trajectory. Deciphering the intricacies of this relationship at the cellular and molecular levels will unveil novel pathways and potential therapeutic targets that could revolutionize wound management and regenerative medicine [3]. This review consolidates our current understanding of wound-healing processes, elucidates the mechanism of ferroptosis, and discusses the latest advancements in understanding their interplay. Furthermore, we discuss promising avenues for regulating ferroptosis to promote healing in both chronic and acute wound environments.

## Review

### Characteristics of ferroptosis

**Figure 1:**
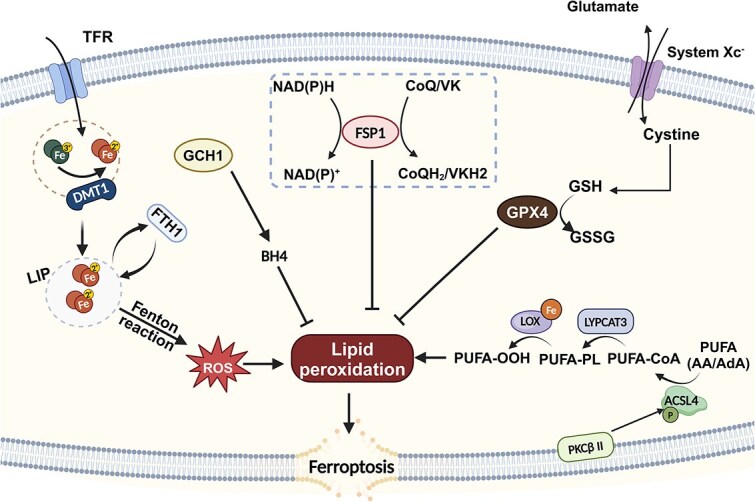
Mechanism of ferroptosis. Ferroptosis is regulated by various metabolic events and signaling pathways, including lipid metabolism, redox-active iron, and the lipid-peroxide repair network. PUFAs such as arachidonic acid (AA) and adrenic acid (AdA) are converted to their CoA derivatives by ACSL4. LPCAT3 incorporates these derivatives into phospholipids, forming PUFA-PL. Subsequently, PUFA-PL is peroxidized by LOX to generate PUFA-OOH, thereby contributing to lipid peroxidation. Cellular iron is another essential factor of ferroptosis; Fe^3+^ is imported into the cell via the TFR1 and is reduced to Fe^2+^ by DMT1. LIP contributes to lipid peroxidation through the Fenton reaction. GPX4, along with GSH, reduces lipid peroxides to their nontoxic forms. FSP1 generates RTAs, including CoQ_10_H_2_ (reduced CoQ) and VKH2 (reduced vitamin K), which neutralize lipid peroxyl radicals. GCH1 produces BH4, a cofactor in antioxidant defense. *PUFAs* polyunsaturated fatty acids, *ACSL4* long-chain fatty acid-CoA ligase 4, *LPCAT3* lysophosphatidylcholine acyltransferase 3, *AA* arachidonic acid, *AdA* adrenic acid, *LOX* lipoxygenases, *TFR1* transferrin receptor 1, *DMT1* divalent metal transporter 1, *LIP* labile iron pool, *GSH* glutathione, *GPX4* glutathione peroxidase 4, *BH4* tetrahydrobiopterin, *VKH2* hydroquinone vitamin K

In 2012, Dixon *et al*. introduced the concept of ferroptosis, a unique form of regulated cell death characterized by iron-dependent lipid peroxidation [4]. Recent research has highlighted its implications in various pathological conditions including IRI, neurodegenerative diseases, acute heart injury, and cancer. Ferroptosis is distinct from apoptosis, autophagy, and necrosis in terms of cellular mechanisms and morphological changes [2]. The morphological features of ferroptosis include preserved integrity of the plasma membrane, mitochondrial shrinkage, and the reduction or disappearance of mitochondrial cristae [5]. The regulation of ferroptosis is intricately linked to lipid metabolism, redox-active iron, and the lipid-peroxide repair network in cells [6] (Figure 1).

#### Lipid metabolism and ferroptosis

Lipid peroxidation is triggered by the oxidation of the carbon–carbon double bonds of polyunsaturated fatty acyl chains within phospholipids (PL-PUFA) in cellular membranes. This process leads to the accumulation of phospholipid hydroperoxides (PLOOHs), which rapidly and irreparably damage the cell membranes, culminating in cell death [7]. Two enzymes, long-chain fatty acid-coenzyme A (CoA) ligase 4 (ACSL4) and lysophosphatidylcholine acyltransferase 3 (LPCAT3), are crucial for PL-PUFA production. ACSL4 catalyzes the conjugation of CoA with arachidonic acid (20:4) and adrenic acid (22:4), critical polyunsaturated fatty acids (PUFAs) that induce ferroptosis [8, 9]. Subsequently, LPCAT3 esterifies these CoA-bound acids to phospholipids, enhancing the incorporation of long-chain PUFAs into the lipid and membrane structure [10]. Furthermore, protein kinase C beta II (PKCβII) phosphorylates and activates ACSL4, acting as a lipid peroxidation sensor and promoting a positive feedback loop that accelerates lipid peroxidation [11]. The balance between PUFAs and monounsaturated fatty acids (MUFAs) is important for maintaining lipid peroxidation levels. Endogenous production of PL-MUFA by enzymes such as stearoyl-CoA desaturase 1 (SCD1) and ACSL3 or exogenous supplementation of PL-MUFA can inhibit ferroptosis [12]. Deuterated polyunsaturated fatty acids (D-PUFAs), which prevent the formation of PL-PUFAs, have been used to protect cells and tissues from ferroptosis [13]. Additionally, polyunsaturated ether phospholipids (ePL-PUFAs), synthesized in peroxisomes, serve as substrates for lipid peroxidation, contributing to the resistance of cancer cells to ferroptosis [14]. Recently, MBOAT1/2 was found to inhibit ferroptosis by altering the cellular phospholipid profile [15].

#### Iron homeostasis and ferroptosis

Iron plays a crucial role in ferroptosis because iron chelators can completely inhibit this process. Iron promotes lipid peroxidation via both enzyme-dependent and enzyme-independent Fenton reactions. Under acidic conditions, iron reacts with peroxides to generate the free radical PLO•. This initiates a damaging chain reaction, producing further radicals such as PLOO•, which escalates lipid peroxidation and eventually leads to cell death [7]. Additionally, several redox enzymes, including lipoxygenases (LOXs) and cytochrome P450 oxidoreductase (POR), which are key regulators of ferroptosis, require iron for their catalytic activity [9, 16, 17]. Therefore, maintaining iron homeostasis is critical for preventing ferroptosis.

Cellular iron homeostasis is primarily governed by the iron sensor proteins iron-responsive element-binding protein 1 (IRP1) and iron-responsive element-binding protein 2 (IRP2), which regulate the expression of genes essential for iron uptake, storage, and export [18]. Extracellular iron bound to transferrin is internalized by cells via transferrin receptor 1 (TFR1). Inside the endosome, iron (Fe^3+^) is dissociated from transferrin and reduced to iron (Fe^2+^), which is subsequently transported into the cytoplasm by divalent metal transporter 1 (DMT1), contributing to the labile iron pool (LIP) [18]. LIP is crucial not only for the synthesis of mitochondrial proteins, such as iron–sulfur clusters, but also for iron storage in ferritin. Ferritin-bound iron can be mobilized back into the LIP via ferritinophagy [19].

Ferritin heavy chain 1 (FTH1) prevents ferroptosis in cardiomyocytes, and it is vital for the activity of leukemic hematopoietic stem cells [20, 21]. Adin *et al*. discovered that disrupting the interaction between TFR1 and transferrin decreases the iron content in vascular smooth muscle cells, inhibits iron-dependent oxidative reactions, and delays the onset of abdominal aortic aneurysm [22]. Furthermore, studies have shown that dihydroartemisinin increases the LIP content by binding to free cellular iron and activating IRPs, thereby enhancing the sensitivity of cancer cells to ferroptosis [23].

#### Lipid-peroxide repair network and ferroptosis

Cells have various lipid-peroxide repair networks that counteract lipid peroxidation and prevent ferroptosis. These systems include the glutathione peroxidase 4 (GPX4)–glutathione (GSH) system, nicotinamide adenine dinucleotide phosphate (NAPDH)–ferroptosis suppressor protein 1 (FSP1)–coenzyme Q10 (CoQ_10_)/vitamin K system, GTP cyclohydrolase 1 (GCH1)–tetrahydrobiopterin (BH4) system, and other antioxidant mechanisms [24]. GPX4 is a critical enzyme within the glutathione peroxidase family and the only known enzyme capable of directly reducing PLOOHs in cells [25, 26]. The cystine/glutamate antiporter system Xc−, which consists of the SLC7A11 and SLC3A2 subunits, plays a vital role by importing cystine into cells [4]. Once inside, cystine is converted via a series of enzymatic reactions to synthesize GSH. GSH serves as a substrate for GPX4 and participates in the reduction of lipid peroxides, through which it is converted to oxidized GSH. Compounds that inhibit either GPX4 or the system Xc− significantly contribute to the onset of ferroptosis.

FSP1 is the second most critical suppressor of ferroptosis. Serving as an NADPH-ubiquinone reductase, FSP1 converts CoQ_10_ into its reduced form CoQ_10_H_2_, which acts as an endogenous radical-trapping antioxidant (RTA) that reduces lipid peroxides [27, 28]. Recent studies have shown that FSP1 is involved in the noncanonical vitamin K pathway, producing a reduced form of vitamin K that helps inhibit lipid peroxidation and ferroptosis [29]. Considering the critical role of GPX4 in maintaining cell survival in various healthy tissues, targeting this enzyme may lead to severe adverse effects. Consequently, FSP1 has emerged as a favorable target for cancer therapies [30]. The development of icFSP1, the first described on-target FSP1 inhibitor that promotes FSP1 phase separation, has shown potential for sensitizing cancer cells to ferroptosis and hindering tumor growth [31].

GCH1 synthesizes dihydrobiopterin/tetrahydrobiopterin (BH_2_/BH_4_), both of which can counteract lipid peroxides [32]. Dihydroorotate dehydrogenase (DHODH) mediates ferroptosis defense mechanisms in the mitochondria by reducing ubiquinone to ubiquinol [33]. Additionally, in the final step of cholesterol biosynthesis, 7-dehydrocholesterol reductase (DHCR7) consumes its substrate 7-dehydrocholesterol (7-DHC), which shields lipids from autoxidation and subsequent fragmentation, potentially enabling cancer cells to evade ferroptosis [34, 35]. Lipid-peroxide repair networks serve as antagonists of ferroptosis, shielding both normal and cancerous tissues from this form of cell death. These networks offer various targets for therapeutic interventions under different pathological conditions.

#### Pathological and physiological role of ferroptosis

Ferroptosis is a key factor influencing the progression of various diseases. Ischemia reperfusion induces massive oxidative stress, leading to damage in multiple organs, including the brain, liver, and kidneys [36]. Extensive studies have shown that ferroptosis plays a critical role in the cell death and inflammation associated with IRI. Ferroptosis is implicated in neurodegenerative diseases such as Huntington’s disease (HD), Alzheimer’s disease (AD), Parkinson’s disease (PD), and amyotrophic lateral sclerosis (ALS) [37]. Ferroptosis inhibitors such as the iron chelators deferiprone (DFP) and ferrostatin-1 (Fer-1) have been reported to alleviate these conditions in mouse models. Furthermore, ferroptosis contributes to organ injuries associated with nonalcoholic steatohepatitis (NASH) [38], doxorubicin (DOX)-induced cardiomyopathy [20], and chronic obstructive pulmonary disease associated with cigarette smoking [39].

The physiological role of ferroptosis is a focal point in this field. In the immune system, T follicular helper cell (Tfh) homeostasis is regulated by the selenium-GPX4-ferroptosis axis, and selenium supplementation enhances Tfh cell function during infections and postvaccination [40]. Additionally, ferroptosis is induced in tumors via a mechanism where T-cell-derived IFN-γ combines with arachidonic acid in the microenvironment, functioning as a pathway for CD8^+^T-cell-mediated tumor elimination [41]. Ferroptosis also plays a pivotal role in the developmental cell death of the rice blast [42]. Recent studies have demonstrated that ferroptosis can propagate over long distances between cells through reactive oxygen species (ROS)–triggered waves, underscoring its role in coordinating global cell death events during muscle remodeling in the embryonic avian limb [43]. Furthermore, the impact of ferroptosis on aging has been documented in the nematode *Caenorhabditis elegans* [44].

The last decade has seen rapid progress in research on ferroptosis [2]. On one hand, unveiling the regulatory mechanisms underlying ferroptosis may pave the way for the development of potent therapeutic approaches targeting this process. On the other hand, elucidating the physiological and pathological roles of ferroptosis has significantly deepened our understanding of its implications across multiple diseases, including wound healing, as discussed further below.

### Ferroptosis in wound healing

**Figure 2:**
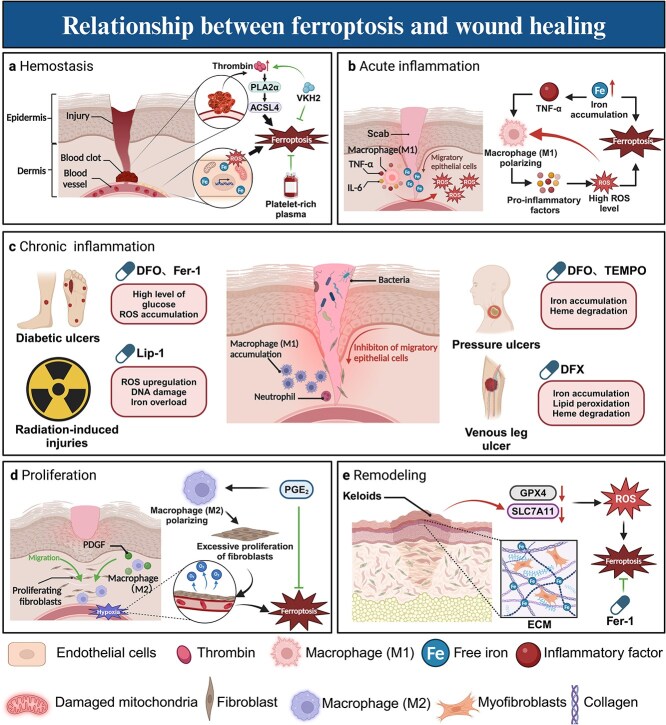
Relationship between wound healing and ferroptosis. (**a**) Hemostasis is characterized by the activation of thrombin, which can induce ferroptosis. During this phase, endothelial cells may also be susceptible to ferroptosis. Importantly, both platelet-rich plasma and the procoagulant factor VKH2 have been shown to inhibit the occurrence of ferroptosis. (**b**) During the acute inflammatory phase of wound healing, macrophages polarize into M1 macrophages, which secrete pro-inflammatory cytokines and produce ROS. Concurrently, a notable accumulation of iron occurs at the site of injury. The interaction among iron, ROS, inflammatory mediators, and the sustained polarization of macrophages establishes a significant relationship with ferroptosis. (**c**) Chronic inflammation is characterized by the persistent polarization of M1 macrophages, which play a pivotal role in conditions such as diabetic ulcers, radiation injuries, pressure ulcers, and lower extremity venous ulcers. Inhibitors of ferroptosis have been shown to mitigate chronic inflammation and different types of chronic wounds. (**d**) During the proliferation phase, the migration and rapid proliferation of fibroblasts may induce localized hypoxia, which subsequently triggers ferroptosis. In this context, PEG2 may serve as a potential suppressor of ferroptosis. (**e**) During the remodeling phase, myofibroblasts influence ECM components, leading to scar formation. The levels of ferroptosis suppressors such as GPX4 and SLC7A11 are significantly reduced in hypertrophic scars. Fer-1 suppresses scar formation by inhibiting ferroptosis. *ROS* reactive oxygen species, *PEG2* prostaglandin E2, *SLC7A11* solute carrier family 7 member 11, *GPX4* glutathione peroxidase 4, *ECM* extracellular matrix

Tissue wound healing constitutes a multifaceted phenomenon that encompasses various biological processes and cell types that collaborate to restore the structure and function of injured tissue [45]. This intricate process occurs in four sequential stages: hemostasis, inflammation, proliferation, and remodeling [46]. Each of these stages is pivotal in the overall repair process, necessitating the meticulous coordination of diverse cellular signals and biochemical pathways. Remarkably, ferroptosis has been implicated in these stages and in various types of wound healing (Figure 2).

#### Hemostasis

The primary objective at injury sites is to quickly seal the exposed tissue, which prevents fluid loss and impedes pathogen invasion. Hemostasis is the initial stage of wound healing and is characterized by the severing of blood vessels at the injury site. This process results in damage to epidermal and connective tissue cells, as well as disruption of the extracellular matrix (ECM) [47]. Following injury, vasoconstriction occurs to reduce blood loss, followed by the release of adenosine diphosphate from endothelial cells to attract platelets. Subsequently, platelets adhere to the damaged vessel wall, become activated, and aggregate to form a clot. This clot blocks the damaged vessel, effectively sealing it. Subsequently, the coagulation cascade is activated, leading to the formation of thrombin, which converts fibrinogen into an insoluble fibrin network, culminating in the formation of a blood clot [48]. During the final stage of clotting, the surface of the blood clot undergoes dehydration and forms a scab that effectively prevents further fluid loss. Consequently, the damaged blood vessels are sealed, and bleeding is halted. Studies have shown that targeted delivery of extracellular vesicle–derived nanoparticles containing milk fat globule EGF factor 8 (MFGE8) to endothelial cells can promote wound healing by counteracting ferroptosis induced by mitochondrial damage [49]. In the context of diabetic ulcers, the inhibition of ferroptosis in endothelial cells facilitates wound healing [50]. Studies have found that platelets can inhibit ferroptosis and promote wound healing. For example, platelet-rich plasma has been found to inhibit ferroptosis and promote the healing of diabetic ulcers; however, the exact mechanism of this process remains to be fully elucidated [51]. In addition, platelet-rich fibrin significantly promotes wound healing [52]. Because of the procoagulant role of thrombin, it has been incorporated in several wound-healing materials [53, 54]. Vitamin K hydroquinone (VKH2), a coagulation promoter that facilitates thrombin generation, inhibits lipid peroxidation and prevents ferroptosis. Both Vitamin K Epoxide Reductase Complex Subunit 1 Like 1 (VKORC1L1) and FSP1 reduce vitamin K to VKH2, thereby facilitating coagulation and suppressing ferroptosis [29]. However, some studies have suggested that fibrin matrices containing high concentrations of thrombin may trigger inflammation and delay wound healing compared with matrices with lower thrombin concentrations [55]. In cerebral IRI and cancer, increased thrombin levels have been found to activate the PLA2α–ACSL4 pathway, which induces ferroptosis [56, 57]. Ferroptosis may significantly affect platelet activation during the coagulation process. Under oxidative stress, hemoglobin releases heme, a cofactor that catalyzes ROS generation. This pathway may modulate the activation of human platelets via ferroptosis [58].

#### Inflammation

##### Acute inflammation

After completion of the hemostasis stage, the inflammation stage begins promptly. The cellular response during the inflammatory stage is characterized by an influx of leukocytes into the wound area. Within the blood clot, monocytes differentiate into macrophages that recruit neutrophils to the wound site [59]. These M1-polarized macrophages and neutrophils play a critical role in eliminating bacteria through an oxygen-dependent pathway and in clearing the wound by engulfing dead bacteria and cellular debris [60]. Although several studies have revealed the necessity of inflammation in wound cleansing, an excessive inflammatory response can lead to increased collagen deposition and the formation of scars [61, 62]. During this process, leukocytes secrete various pro-inflammatory factors such as TNF-α, IL-1β, IL-6, and IFN-γ. These cytokines further trigger ROS production, amplifying the oxidative–reductive reactions at the site of inflammation and creating an inflammatory cycle [63]. The relationship between iron, ROS, inflammatory factors, and the sustained polarization of macrophages is complex and intricate. The accumulation of iron within macrophages primarily stems from heme iron obtained through the phagocytosis of erythrocytes, as well as nonheme iron released from damaged or dead cells. On one hand, iron accumulation in M1 macrophages significantly enhances the secretion of TNFα *in vitro*, thereby further promoting the polarization of macrophages into the M1 phenotype through the NF-kB signaling pathway [64, 65]. A similar phenomenon has been observed in venous ulcers [66]. Additionally, iron accumulation in macrophages can drive polarization by increasing ROS production and inducing p53 acetylation [66–68]. On the other hand, pro-inflammatory cytokines such as IL-6 can influence macrophage iron metabolism by regulating systemic iron homeostasis. Both iron chelators and antioxidants have been shown to facilitate wound healing [69, 70], which suggests their potential therapeutic benefits. Considering that both ROS and iron are critical triggers of ferroptosis and that ferroptosis tends to exacerbate the inflammatory response, further exploration of the role of ferroptosis during this stage of wound healing is important. Ferroptosis has been implicated in the dysregulation of this stage, which results in chronic inflammation.

##### Chronic inflammation

When the inflammatory response is persistently activated during wound healing, failure of M1 macrophages to transition to the M2 phenotype can lead to the formation of chronic wounds [71]. Chronic wounds are characterized by their inability to heal within the expected timeframe. These wounds arise from various factors such as diabetic hyperglycemia, infections, and radiation. They exhibit elevated levels of ROS, inflammatory cytokines, and bacteria [72]. Below, we summarize the relationship between different types of chronic wounds and ferroptosis.

###### Diabetic ulcers

Diabetes is a prevalent chronic disease characterized by hyperglycemia. Diabetic foot ulcers are common and highly morbid complications of diabetes that lead to a decline in functional status, infections, hospitalizations, lower-extremity amputations, and death [73]. High iron levels are a risk factor for diabetes, and they contribute to diabetic phenotypes in various tissues [74]. Additionally, cells in hyperglycemic environments exhibit elevated ROS levels, which can cause oxidative damage and cell death [75, 76]. Iron targeting in diabetic ulcers has been shown to promote wound healing. Transdermal delivery of deferoxamine (DFO) prevents diabetic ulcer formation and enhances the healing of existing diabetic wounds [77]. Moreover, DFO released from hydrogel nanofibrous scaffolds upregulates the expression of HIF-1α, which leads to the rapid recruitment of angiogenesis-related cells and wound healing in diabetic rats [78]. Secretory autophagosomes reduce the production and release of free iron from human dermal fibroblasts in high-glucose environments, thereby improving diabetic wound healing [79]. Interestingly, ROS delays diabetic wound healing by triggering ferroptosis, and inhibition of ferroptosis with Fer-1 has been found to accelerate wound healing in diabetic rats [80]. Furthermore, research has demonstrated that the anti-ferroptotic and anti-inflammatory effects of Fer-1, which promote diabetic wound healing, are mediated through the stimulation of nuclear erythroid 2-related factor 2 (Nrf2) [81].

###### Radiation-induced injuries

Since its discovery, ionizing radiation has been a pivotal component of cancer therapy. Despite its therapeutic benefits, ionizing radiation induces ROS production and DNA breaks in normal tissues, leading to significant organ damage and skin injury [82]. Recent advances in ferroptosis have shown that irradiation induces ferroptosis through both ROS generation and ACSL4 upregulation [83, 84]. Targeting ferroptosis mitigates irradiation-induced injury in various tissues [85–87]. Ultraviolet (UV) irradiation induces lipid peroxide accumulation and iron overload, leading to keratinocyte death via ferroptosis and skin inflammation [88]. Nicotinamide mononucleotide (NMN) and liproxstatin-1 (Lip-1) strongly mitigate UV-induced skin injury via defense ferroptosis [89].

###### Pressure ulcers

Pressure ulcers, caused by sustained pressure, is typically characterized by persistent necrotic tissue and abnormal iron accumulation. Recent studies have shown that the knockout of myoglobin (a protein composed of globin and ferric heme) or administration of DFO can lower the levels of oxidative stress markers and alleviate tissue damage at pressure ulcer sites [90]. Targeting endothelial cells with extracellular vesicle–derived nanoparticles containing MFGE8 mitigates ferroptosis, facilitating the healing of pressure ulcers [49]. Furthermore, the antioxidant 2,2,6,6-tetramethylpiperidine-1-oxyl (TEMPO) substantially decreases pressure ulcer formation following skin ischemia–reperfusion (I/R), primarily because of its inhibitory effect on ferroptosis [91].

###### Venous leg ulcers

Chronic venous diseases frequently result in skin pigmentation and leg ulcers. Research has demonstrated that varicose veins in the legs can lead to the accumulation of red blood cells, and the subsequent breakdown of hemoglobin results in excess tissue iron. Iron deposition on the skin is a hallmark of leg ulcers [92]. Furthermore, studies have shown that iron accumulation in chronic venous leg ulcers is accompanied by elevated lipid peroxidation [93, 94]. The iron chelator DFX has been found to significantly promote wound healing [66], indicating the involvement of ferroptosis in chronic venous ulcers.

In addition to chronic wound healing, ferroptosis plays a significant role in traumatic neuronal injuries, including controlled cortical impact injury, traumatic spinal cord injury, and traumatic hippocampal injury [95–97]. Both intraventricular injection of Fer-1 and GPX4 overexpression have been shown to significantly reduce iron accumulation and neuronal damage by inhibiting ferroptosis [95, 98, 99].

Typical chronic wounds exhibit a notable accumulation of iron and ROS, which leads to significant ferroptosis. The application of ferroptosis inhibitors markedly reduces inflammation and expedites wound healing. The link between chronic wounds and ferroptosis is becoming increasingly evident, highlighting the potential of therapeutic interventions targeting ferroptosis to improve the outcomes of patients with chronic wounds.

#### Proliferation

The proliferation stage of wound healing involves the formation of new cells and tissues. It is involved in the proliferation and migration of fibroblasts and epithelial cells and in angiogenesis. Epithelial cells at the wound edge migrate inward, covering the wound and restoring the epithelium through a process called re-epithelialization [100]. Concurrently, after an injury and during the resolution of the inflammatory response, fibroblasts begin to migrate from the surrounding dermis to the blood clot. The granulation tissue, stimulated by cytokines released by M2 polarized macrophages such as PDGF, TGF-β, EGF, and IGF-1, replaces the fibrin clot and rapidly generates a new ECM [101]. Prostaglandin E2 (PGE_2_) modulates the repair of various tissues through activation of stem cells, inflammation, and angiogenesis [102]. During the proliferation stage of cutaneous wound healing, PGE_2_ improves M2 macrophage polarization [103]. Interestingly, PGE2 signaling protects cells from DOX-induced cardiomyocyte ferroptosis and ferroptosis induced by cerebral I/R [104, 105].

**Table 1:** Commonly utilized ferroptosis inhibitors

**Class**	**Drugs**	**Target**	**Animal models**	**Clinical development**	**Application in wound healing**
Synthetic RTAs	Fer-1	Lipid peroxidation, 15Lox/PEBP1	Huntington’s disease, iron-overload-induced liver damage, and DOX-induced cardiotoxicity	N/A	Diabetic wounds [80, 81], traumatic brain injury [95, 98, 99], fibrosis in keloids [118]
	Lip-1	Lipid peroxidation	GPX4 KO mice; liver IRI	N/A	UV-induced skin injury [89]
	TEMPO	Lipid peroxidation	Cardiac reperfusion, brain reperfusion	N/A	Pressure ulcers [91]
	CuATSM	Lipid peroxidation	N/A	Parkinson’s disease; amyotrophic lateral sclerosis	N/A
Endogenous lipophilic RTAs	CoQ_10_	Lipid peroxidation	Cancer cell sensitivity to ferroptosis	N/A	N/A
	BH4	Lipid peroxidation	Cancer cell sensitivity to ferroptosis	N/A	N/A
	7-DHC	Lipid peroxidation	Renal IRI	N/A	N/A
Natural RTAs	Silibinin	Lipid peroxidation	Renal IRI; GPX4-KO-induced liver and renal injury	Health product for liver protection	N/A
	Vitamin E	Lipid peroxidation	GPX4 KO in endothelial cells, liver, and hematopoietic system	Daily nutrition	N/A
	Vitamin K	Lipid peroxidation	GPX4 KO in liver, liver, or kidney IRI	Daily nutrition	N/A
Iron chelators	DFO	Labile iron	Neurodegeneration, IRI, NASH, and hemorrhagic stroke	Iron-overload-related diseases	Diabetic wounds [77, 78], pressure ulcers [90], traumatic spinal cord injury [95]
	DFP	Labile iron	Iron-overload-induced ferroptosis in cerebral toxoplasmosis, rhabdomyolysis-induced acute kidney injury	Parkinson’s disease, Frederich’s ataxia	N/A
Selenium	Tat SelPep	GPX4	Neuronal IRI	Nutrition element	N/A
Thiazolidinediones	Pioglitazone	ACSL4	Intracerebral hemorrhage	Type 2 diabetes	N/A
	Rosiglitazone	ACSL4	GPX4 KO mice, intestinal IRI	Type 2 diabetes	N/A
D-PUFAs	RT001	PUFA autoxidation	Neurodegeneration	Friedreich’s ataxia; infantile neuroaxonal dystrophy	N/A

*PEBP1* phosphatidylethanolamine binding protein 1, *Lip-1* liproxstatin-1, *RTAs* radical-trapping antioxidants, *DFO* deferoxamine, *DFP* deferiprone, *ACSL4* long-chain fatty acid-CoA ligase 4, *PUFA* polyunsaturated fatty acid, *DOX* doxorubicin, *GPX4* glutathione peroxidase 4, *TEMPO* 2,2,6,6-tetramethylpiperidine-1-oxyl, *Fer-1* ferrostatin-1, *IRI* ischemia–reperfusion injury, *N/A* not applicable

During the proliferative phase, the proliferation of newly formed capillaries and fibroblasts promotes the formation of granulation tissue. However, the rapid proliferation of fibroblasts may create a localized ischemic and hypoxic microenvironment [106]. Studies have shown that oxygen and glucose deprivation in primary mouse skin fibroblasts induces ferritinophagy mediated by nuclear receptor coactivator 4 (NCOA4), leading to ferroptosis. In mouse models of ischemic wounds, the inhibition of both ferroptosis and autophagy promotes wound healing [107]. Endothelial cells play a crucial role in promoting capillary formation, maintaining vascular integrity, and regulating tissue remodeling. High glucose concentrations enhance ferroptosis in endothelial cells by activating the p53-SLC7A11-GSH axis [108]. Moreover, both endothelial cells and fibroblasts under high-glucose conditions exhibit increased lipid peroxidation levels, upregulation of ferroptosis-related proteins, and reduced cell survival and migration capacity. Notably, Fer-1 reverses these effects and promotes wound healing in diabetic rats [80].

#### Remodeling

In the final stage of wound healing, the granulation tissue undergoes transformation into scar tissue, which involves the synthesis and degradation of collagen and ECM components [109]. A key aspect of this process is the activity of matrix metalloproteinases secreted by epidermal cells, which play a central role in tissue remodeling [110, 111]. During the remodeling process, the population of inflammatory cells and fibroblasts within the wound area substantially decreases. Because of these cellular changes, scars are formed with a reduced number of cells [112]. Fibroblasts synthesize and secrete collagen, which contributes to the reconstruction and reinforcement of tissue structure in the wound area [113, 114]. If fibroblasts abnormally proliferate and differentiate into myofibroblasts, excessive collagen-rich ECM is produced, leading to fibrosis and ultimately keloid formation [115]. RNA sequencing has revealed differentially expressed ferroptosis-related genes in keloid tissues compared to those in normal human skin [116]. A therapy against keloid induces ferroptosis in keloid fibroblasts via ROS, accompanied by downregulation of SLC7A11 and GPX4 [117]. Additionally, iron has been demonstrated to accumulate in keloid tissue, along with significant downregulation of SLC7A11, GPX4, and Nrf2 and upregulation of transferrin receptor protein 1 (TFRC). Fer-1 inhibits ferroptosis, thereby reducing ECM deposition and fibrosis [118]. In addition, the genetic deletion of ferroportin, a crucial iron regulator, in macrophages affects stromal cell proliferation, blood and lymphatic vessel formation, and fibrogenesis during wound healing, without affecting inflammatory processes [119].

In summary, ferroptosis regulators play crucial roles at various stages of wound healing. Notably, a positive feedback mechanism involving ROS, iron, and inflammation links wound healing with lipid peroxidation. An imbalance between lipid peroxidation and repair mechanisms triggers ferroptosis and persistent inflammatory response, potentially exacerbating hard-to-heal wounds. Systemic disorders, such as diabetes and immunosuppression, significantly impact ferroptosis regulators during wound healing. Targeting these factors can potentially optimize the healing process and increase the likelihood of successful wound closure.

### Therapeutic implication of ferroptosis in wound healing

Recent studies have focused on the identification and synthesis of ferroptosis inhibitors, which hold significant promise for therapeutic applications in diseases linked to ferroptosis (Table 1). RTAs directly target lipid peroxidation to inhibit ferroptosis. Fer-1, the first synthetic RTA used to inhibit ferroptosis, is effective in various disease models, including HD, iron-overload-induced liver damage, and DOX-induced cardiotoxicity [20, 120, 121]. In addition, Fer-1 has been used to alleviate diabetic wounds, traumatic brain injury, and fibrosis in keloids. However, its low metabolic stability limits its application *in vivo* [122]. Lip-1, which inhibits ferroptosis in UV-induced skin injury, is more suitable for use in animal models than Fer-1 [26]. TEMPO, an organic nitroxyl radical, shares common functions with antioxidants such as Fer-1 but uniquely exhibits volatile effects. Recent research has highlighted the role of TEMPO in mitigating oxidative-stress-related tissue damage following cardiac reperfusion and protecting against ferroptosis-induced cell death after brain reperfusion in mouse models [123]. Additionally, it reduces the progression of cutaneous IRI to pressure ulcer formation [91]. Copper (II)-diacetyl-bis(*N*^4^-methylthiosemicarbazone) (CuATSM) and its structurally similar analog CuATSP are other notable synthetic RTAs, with CuATSM demonstrating potent antiferroptotic activities in clinical settings for neurodegenerative diseases such as ALS and PD [124].

FSP1 has been identified as a crucial ferroptosis suppressor by reducing endogenous lipophilic RTA coenzyme Q10 and natural lipophilic RTA vitamin K, thereby reducing lipid peroxidation and subsequent ferroptosis [27–29]. BH4 is an important RTA that, alone or in conjunction with vitamin E, shields lipid membranes from oxidative damage, further underpinning cellular defense mechanisms against ferroptosis [32, 125]. Recently, 7-DHC, an intermediate metabolite in distal cholesterol biosynthesis, was identified as an endogenous inhibitor of ferroptosis [34, 35]. Enhancing 7-DHC levels by inhibiting DHCR7 has shown potential for managing cancer metastasis and reducing the progression of kidney IRI [34]. Silibinin, a natural product renowned for its hepatoprotective properties, is safe and effective in inhibiting ferroptosis at both cellular and tissue levels [126, 127]. α-Tocopherol, the most active component of vitamin E, is a well-recognized lipophilic RTA. It works synergistically with GPX4 to inhibit ferroptosis in various tissues, including the vascular endothelium, liver, and hematopoietic system [128–131].

Iron chelators such as DFO and DFP sequester iron, thereby preventing the accumulation of iron-dependent lipid peroxides and offering a strategic approach to combat ferroptosis. DFO was the first U.S. Food and Drug Administration (FDA)-approved treatment for diseases associated with iron overload. It attenuates ferroptosis-related diseases in mouse models, including neurodegeneration, NASH, and hemorrhagic stroke. Furthermore, DFO has been shown to alleviate diabetic wounds and traumatic spinal cord injury. However, this may result in side effects such as anemia and edema [132]. DFP, developed as an alternative to DFO, inhibits iron overload induced by cerebral and rhabdomyolysis-induced acute kidney injury [133, 134]. It offers a safer and more cost-effective alternative with enhanced efficacy in clinical settings, such as in neuronal diseases [135].

In addition to ferroptosis inhibitors, therapeutic strategies aimed at modulating ferroptosis include GPX4 activation through selenium supplementation [136]; inhibition of lipoxygenases; and targeting of ACSL4 using drugs such as pioglitazone and rosiglitazone, which are commonly used for their modulatory effect on peroxisome proliferator–activated receptor gamma (PPARγ) in diabetes management [8]. Moreover, clinical trials involving D-PUFAs to block the biosynthesis of pro-oxidative phospholipids have underscored their therapeutic potential in neurodegenerative diseases [137, 138]. Therefore, it is worthwhile to apply these clinically applicable inhibitors of ferroptosis for wound healing.

Wound healing involves intricate cellular processes in which ferroptosis plays a significant role. By specifically targeting ferroptosis at wound sites, pharmacological interventions can potentially reduce systemic side effects while maximizing local therapeutic efficacy. Furthermore, the integration of advanced wound dressings such as hydrocolloids, foam, alginate, and hydrogels optimizes healing conditions, offering significant prospects for the development of novel therapeutic strategies that improve recovery outcomes in both chronic and acute wound scenarios.

## Conclusions

Ferroptosis modulators play important roles throughout the different stages of wound healing. The use of ferroptosis inhibitors is a groundbreaking approach for wound management. Wound healing is a complex process involving numerous tissues and cell types and is characterized by a distinct temporal sequence. However, a comprehensive panoramic map depicting its initiation and progression is lacking. Most existing studies have focused on individual cell types, or they have been limited to phenotypic analyses, rendering the identity of the cell types undergoing ferroptosis at different stages ambiguous. Therefore, harnessing technologies such as single-cell transcriptomics and spatial omics is essential for comprehensively mapping these processes. Precision medicine and targeted therapies are currently used for clinical treatment. Such insights could provide a foundation for developing precision treatments to regulate ferroptosis and potentially facilitate the development of more effective treatment strategies for wound healing. In addition to ferroptosis, other forms of cell death, including necroptosis and apoptosis, are involved in wound healing. Therefore, combined approaches targeting different types of cell death may offer a novel therapeutic strategy for wound healing.

## References

[1] Almadani YH, Vorstenbosch J, Davison PG, Murphy AM. Wound healing: a comprehensive review. Semin Plast Surg 2021;35:141–4. 10.1055/s-0041-1731791. 34526860 PMC8432991

[2] Stockwell BR . Ferroptosis turns 10: emerging mechanisms, physiological functions, and therapeutic applications. Cell 2022;185:2401–21. 10.1016/j.cell.2022.06.003. 35803244 PMC9273022

[3] Bi M, Li D, Zhang J. Research progress and insights on the role of ferroptosis in wound healing. Int Wound J 2023;20:2473–81. 10.1111/iwj.14102. 36788729 PMC10333008

[4] Dixon SJ, Lemberg KM, Lamprecht MR, Skouta R, Zaitsev EM, Gleason CE. et al. Ferroptosis: an iron-dependent form of nonapoptotic cell death. Cell 2012;149:1060–72. 10.1016/j.cell.2012.03.042. 22632970 PMC3367386

[5] Xie Y, Hou W, Song X, Yu Y, Huang J, Sun X. et al. Ferroptosis: process and function. Cell Death Differ 2016;23:369–79. 10.1038/cdd.2015.158. 26794443 PMC5072448

[6] Stockwell BR, Jiang X. The chemistry and biology of Ferroptosis. Cell Chem Biol 2020;27:365–75. 10.1016/j.chembiol.2020.03.013. 32294465 PMC7204503

[7] Conrad M, Pratt DA. The chemical basis of ferroptosis. Nat Chem Biol 2019;15:1137–47. 10.1038/s41589-019-0408-1. 31740834

[8] Doll S, Proneth B, Tyurina YY, Panzilius E, Kobayashi S, Ingold I. et al. ACSL4 dictates ferroptosis sensitivity by shaping cellular lipid composition. Nat Chem Biol 2017;13:91–8. 10.1038/nchembio.2239. 27842070 PMC5610546

[9] Kagan VE, Mao G, Qu F, Angeli JP, Doll S, Croix CS. et al. Oxidized arachidonic and adrenic PEs navigate cells to ferroptosis. Nat Chem Biol 2017;13:81–90. 10.1038/nchembio.2238. 27842066 PMC5506843

[10] Reed A, Ichu TA, Milosevich N, Melillo B, Schafroth MA, Otsuka Y. et al. LPCAT3 inhibitors remodel the polyunsaturated phospholipid content of human cells and protect from Ferroptosis. ACS Chem Biol 2022;17:1607–18. 10.1021/acschembio.2c00317. 35658397

[11] Zhang HL, Hu BX, Li ZL, Du T, Shan JL, Ye ZP. et al. PKCβII phosphorylates ACSL4 to amplify lipid peroxidation to induce ferroptosis. Nat Cell Biol 2022;24:88–98. 10.1038/s41556-021-00818-3. 35027735

[12] Magtanong L, Ko PJ, To M, Cao JY, Forcina GC, Tarangelo A. et al. Exogenous monounsaturated fatty acids promote a Ferroptosis-resistant cell state. Cell Chem Biol 2019;26:420–432.e9. 10.1016/j.chembiol.2018.11.016. 30686757 PMC6430697

[13] Yang WS, Kim KJ, Gaschler MM, Patel M, Shchepinov MS, Stockwell BR. Peroxidation of polyunsaturated fatty acids by lipoxygenases drives ferroptosis. Proc Natl Acad Sci USA 2016;113:E4966–75. 10.1073/pnas.1603244113. 27506793 PMC5003261

[14] Zou Y, Henry WS, Ricq EL, Graham ET, Phadnis VV, Maretich P. et al. Plasticity of ether lipids promotes ferroptosis susceptibility and evasion. Nature 2020;585:603–8. 10.1038/s41586-020-2732-8. 32939090 PMC8051864

[15] Liang D, Feng Y, Zandkarimi F, Wang H, Zhang Z, Kim J. et al. Ferroptosis surveillance independent of GPX4 and differentially regulated by sex hormones. Cell 2023;186:2748–64.e22. 10.1016/j.cell.2023.05.003. 37267948 PMC10330611

[16] Zou Y, Li H, Graham ET, Deik AA, Eaton JK, Wang W. et al. Cytochrome P450 oxidoreductase contributes to phospholipid peroxidation in ferroptosis. Nat Chem Biol 2020;16:302–9. 10.1038/s41589-020-0472-6. 32080622 PMC7353921

[17] Yan B, Ai Y, Sun Q, Ma Y, Cao Y, Wang J. et al. Membrane damage during Ferroptosis is caused by oxidation of phospholipids catalyzed by the oxidoreductases POR and CYB5R1. Mol Cell 2021;81:355–69.e10. 10.1016/j.molcel.2020.11.024. 33321093

[18] Galy B, Conrad M, Muckenthaler M. Mechanisms controlling cellular and systemic iron homeostasis. Nat Rev Mol Cell Biol 2024;25:133–55. 10.1038/s41580-023-00648-1. 37783783

[19] Hou W, Xie Y, Song X, Sun X, Lotze MT, Zeh HJ, 3rd. et al. Autophagy promotes ferroptosis by degradation of ferritin. Autophagy 2016;12:1425–8. 10.1080/15548627.2016.1187366. 27245739 PMC4968231

[20] Fang X, Wang H, Han D, Xie E, Yang X, Wei J. et al. Ferroptosis as a target for protection against cardiomyopathy. Proc Natl Acad Sci USA 2019;116:2672–80. 10.1073/pnas.1821022116. 30692261 PMC6377499

[21] Yi W, Zhang J, Huang Y, Zhan Q, Zou M, Cheng X. et al. Ferritin-mediated mitochondrial iron homeostasis is essential for the survival of hematopoietic stem cells and leukemic stem cells. Leukemia 2024;38:1003–18. 10.1038/s41375-024-02169-y. 38402368

[22] Zhang F, Li K, Zhang W, Zhao Z, Chang F, Du J. et al. Ganglioside GM3 protects against abdominal aortic aneurysm by suppressing Ferroptosis. Circulation 2024;149:843–59. 10.1161/CIRCULATIONAHA.123.066110. 38018467

[23] Chen G-Q, Benthani FA, Wu J, Liang D, Bian Z-X, Jiang X. Artemisinin compounds sensitize cancer cells to ferroptosis by regulating iron homeostasis. Cell Death Differ 2020;27:242–54. 10.1038/s41418-019-0352-3. 31114026 PMC7205875

[24] Dixon SJ, Olzmann JA. The cell biology of ferroptosis. Nat Rev Mol Cell Biol 2024;25:424–42. 10.1038/s41580-024-00703-5. 38366038 PMC12187608

[25] Yang WS, SriRamaratnam R, Welsch ME, Shimada K, Skouta R, Viswanathan VS. et al. Regulation of ferroptotic cancer cell death by GPX4. Cell 2014;156:317–31. 10.1016/j.cell.2013.12.010. 24439385 PMC4076414

[26] Friedmann Angeli JP, Schneider M, Proneth B, Tyurina YY, Tyurin VA, Hammond VJ. et al. Inactivation of the ferroptosis regulator Gpx4 triggers acute renal failure in mice. Nat Cell Biol 2014;16:1180–91. 10.1038/ncb3064. 25402683 PMC4894846

[27] Doll S, Freitas FP, Shah R, Aldrovandi M, da Silva MC, Ingold I. et al. FSP1 is a glutathione-independent ferroptosis suppressor. Nature 2019;575:693–8. 10.1038/s41586-019-1707-0. 31634899

[28] Bersuker K, Hendricks JM, Li Z, Magtanong L, Ford B, Tang PH. et al. The CoQ oxidoreductase FSP1 acts parallel to GPX4 to inhibit ferroptosis. Nature 2019;575:688–92. 10.1038/s41586-019-1705-2. 31634900 PMC6883167

[29] Mishima E, Ito J, Wu Z, Nakamura T, Wahida A, Doll S. et al. A non-canonical vitamin K cycle is a potent ferroptosis suppressor. Nature 2022;608:778–83. 10.1038/s41586-022-05022-3. 35922516 PMC9402432

[30] Jiang X, Stockwell BR, Conrad M. Ferroptosis: mechanisms, biology and role in disease. Nat Rev Mol Cell Biol 2021;22:266–82. 10.1038/s41580-020-00324-8. 33495651 PMC8142022

[31] Nakamura T, Hipp C, Santos Dias Mourão A, Borggräfe J, Aldrovandi M, Henkelmann B. et al. Phase separation of FSP1 promotes ferroptosis. Nature 2023;619:371–7. 10.1038/s41586-023-06255-6. 37380771 PMC10338336

[32] Kraft VAN, Bezjian CT, Pfeiffer S, Ringelstetter L, Muller C, Zandkarimi F. et al. GTP Cyclohydrolase 1/tetrahydrobiopterin counteract Ferroptosis through lipid Remodeling. ACS Cent Sci 2020;6:41–53. 10.1021/acscentsci.9b01063. 31989025 PMC6978838

[33] Mao C, Liu X, Zhang Y, Lei G, Yan Y, Lee H. et al. DHODH-mediated ferroptosis defence is a targetable vulnerability in cancer. Nature 2021;593:586–90. 10.1038/s41586-021-03539-7. 33981038 PMC8895686

[34] Li Y, Ran Q, Duan Q, Jin J, Wang Y, Yu L. et al. 7-Dehydrocholesterol dictates ferroptosis sensitivity. Nature 2024;626:411–8. 10.1038/s41586-023-06983-9. 38297130 PMC11298758

[35] Freitas FP, Alborzinia H, Dos Santos AF, Nepachalovich P, Pedrera L, Zilka O. et al. 7-Dehydrocholesterol is an endogenous suppressor of ferroptosis. Nature 2024;626:401–10. 10.1038/s41586-023-06878-9. 38297129

[36] Zhang J, Su T, Fan Y, Cheng C, Xu L, LiTian. Spotlight on iron overload and ferroptosis: research progress in female infertility. Life Sci 2024;340:122370. 10.1016/j.lfs.2023.122370. 38141854

[37] Ou M, Jiang Y, Ji Y, Zhou Q, Du Z, Zhu H. et al. Role and mechanism of ferroptosis in neurological diseases. Mol Metab 2022;61:101502. 10.1016/j.molmet.2022.101502. 35447365 PMC9170779

[38] Tsurusaki S, Tsuchiya Y, Koumura T, Nakasone M, Sakamoto T, Matsuoka M. et al. Hepatic ferroptosis plays an important role as the trigger for initiating inflammation in nonalcoholic steatohepatitis. Cell Death Dis 2019;10:449. 10.1038/s41419-019-1678-y. 31209199 PMC6579767

[39] Park E-J, Park Y-J, Lee SJ, Lee K, Yoon C. Whole cigarette smoke condensates induce ferroptosis in human bronchial epithelial cells. Toxicol Lett 2019;303:55–66. 10.1016/j.toxlet.2018.12.007. 30579903

[40] Yao Y, Chen Z, Zhang H, Chen C, Zeng M, Yunis J. et al. Selenium–GPX4 axis protects follicular helper T cells from ferroptosis. Nat Immunol 2021;22:1127–39. 10.1038/s41590-021-00996-0. 34413521

[41] Liao P, Wang W, Wang W, Kryczek I, Li X, Bian Y. et al. CD8(+) T cells and fatty acids orchestrate tumor ferroptosis and immunity via ACSL4. Cancer Cell 2022;40:365–78. 10.1016/j.ccell.2022.02.003. PMC9007863 35216678

[42] Shen QA-O, Liang MA-O, Yang FA-O, Deng YA-O, Naqvi NA-O. Ferroptosis contributes to developmental cell death in rice blast. New Phytol 2020;227:1831–46. 10.1111/nph.16636. 32367535

[43] Co HA-O, Wu CC, Lee YC, Chen SA-O. Emergence of large-scale cell death through ferroptotic trigger waves. Nature 2024;631:654–62. 10.1038/s41586-024-07623-6. PMC11639682 38987590

[44] Jenkins NL, James SA, Salim A, Sumardy F, Speed TP, Conrad M. et al. Changes in ferrous iron and glutathione promote ferroptosis and frailty in aging Caenorhabditis elegans. Elife 2020;9:e56580. 10.7554. 10.7554/eLife.56580 PMC7373428 32690135

[45] Rodrigues M, Kosaric N, Bonham CA, Gurtner GC. Wound healing: a cellular perspective. Physiol Rev 2019;99:665–706. 10.1152/physrev.00067.2017. 30475656 PMC6442927

[46] Eming SA, Martin P, Tomic-Canic M. Wound repair and regeneration: mechanisms, signaling, and translation. Sci Transl Med 2014;6:265sr6. 10.1126/scitranslmed.3009337. 25473038 PMC4973620

[47] Barrientos S, Stojadinovic O, Golinko MS, Brem H, Tomic-Canic M. Growth factors and cytokines in wound healing. Wound Repair Regen 2008;16:585–601. 10.1111/j.1524-475X.2008.00410.x. 19128254

[48] Kattula S, Byrnes JR, Wolberg AS. Fibrinogen and fibrin in Hemostasis and thrombosis. Arterioscler Thromb Vasc Biol 2017;37:E13–21. 10.1161/ATVBAHA.117.308564. 28228446 PMC5324399

[49] Luo L, Zhang H, Zhang S, Luo C, Kan X, Lv J. et al. Extracellular vesicle-derived silk fibroin nanoparticles loaded with MFGE8 accelerate skin ulcer healing by targeting the vascular endothelial cells. J Nanobiotechnology 2023;21:455. 10.1186/s12951-023-02185-7. 38017428 PMC10685683

[50] Lu W, Li XY, Wang ZY, Zhao CB, Li Q, Zhang L. et al. Mesenchymal stem cell-derived extracellular vesicles accelerate diabetic wound healing by inhibiting NET-induced ferroptosis of endothelial cells. Int J Biol Sci 2024;20:3515–29. 10.7150/ijbs.97150. 38993565 PMC11234223

[51] Zhou DL, Liang Q, Ge XY, Xu J. Allogeneic platelet-rich plasma inhibits ferroptosis in promoting wound repair of type 2 diabetic ulcers. Free Radic Biol Med 2024;215 (1873-4596 (Electronic)):37–47. 10.1016/j.freeradbiomed.2024.02.020. 38408545

[52] Long T, Li C, Xu F, Xiao J. Therapeutic efficacy of platelet-rich fibrin on surgical site wound healing in patients undergoing oral carcinoma resection: a meta-analysis. Int Wound J 2024;21:e14386. 10.1111/iwj.14386. 37697485 PMC10784624

[53] Ibne Mahbub MS, Bae SH, Gwon JG, Lee BT, Strukova SM, Dugina Tn Fau-Chistov IV. et al., Decellularized liver extracellular matrix and thrombin loaded biodegradable TOCN/chitosan nanocomposite for hemostasis and wound healing in rat liver hemorrhage model immobilized thrombin receptor agonist peptide accelerates wound healing in mice. Int J Biol Macromol 2023;225:1529–42. 10.1016/j.ijbiomac.202. 36436600

[54] Strukova SM, Dugina TN, Chistov IV, Lange M, Markvicheva EA, Kuptsova S. et al. Immobilized thrombin receptor agonist peptide accelerates wound healing in mice. Clin Appl Thromb Hemost 2001;7:325–9. 10.1177/107602960100700414. 11697718

[55] Gugerell A, Pasteiner W, Nürnberger S, Kober J, Meinl A, Pfeifer S. et al. Thrombin as important factor for cutaneous wound healing: comparison of fibrin biomatrices in vitro and in a rat excisional wound healing model. Wound Repair Regen 2014;22:740–8. 10.1111/wrr.12234. 25231003

[56] Xu S, Tuo QZ, Meng J, Wu XL, Li CL, Lei P. Thrombin induces ferroptosis in triple-negative breast cancer through the cPLA2α/ACSL4 signaling pathway. Transl Oncol 2024;39:101817. 10.1016/j.trano. PMC10652120 37939630

[57] Tuo QZ, Liu Y, Xiang Z, Yan HF, Zou T, Shu Y. et al. Thrombin induces ACSL4-dependent ferroptosis during cerebral ischemia/reperfusion. Signal Transduct Target Ther 2022;77:59. 10.1038/s41392-022-00917-z. PMC8866433 35197442

[58] NaveenKumar SK, SharathBabu BN, Hemshekhar M, Kemparaju K, Girish KS, Mugesh G. The role of reactive oxygen species and Ferroptosis in Heme-mediated activation of human platelets. ACS Chem Biol 2018;13:1996–2002. 10.1021/acschembio.8b00458. 29869870

[59] Krzyszczyk P, Schloss R, Palmer A, Berthiaume F. The role of macrophages in acute and chronic wound healing and interventions to promote pro-wound healing phenotypes. Front Physiol 2018;9:419. 10.3389/fphys.2018.00419. 29765329 PMC5938667

[60] Soliman AM, Barreda DR. Acute inflammation in tissue healing. Int J Mol Sci 2022;24:641. 10.3390/ijms24010641. PMC9820461 36614083

[61] Eming SA, Krieg T, Davidson JM. Inflammation in wound repair: molecular and cellular mechanisms. J Invest Dermatol 2007;127:514–25. 10.1038/sj.jid.5700701. 17299434

[62] Medrado AR, Pugliese LS, Reis SR, Andrade ZA. Influence of low level laser therapy on wound healing and its biological action upon myofibroblasts. Lasers Surg Med 2003;32:239–44. 10.1002/lsm.10126. 12605432

[63] Mittal M, Siddiqui MR, Tran K, Reddy SP, Malik AB. Reactive oxygen species in inflammation and tissue injury. Antioxid Redox Signal 2014;20:1126–67. 10.1089/ars.2012.5149. 23991888 PMC3929010

[64] Chen L, Xiong S, She H, Lin SW, Wang J, Tsukamoto H. Iron causes interactions of TAK1, p21ras, and phosphatidylinositol 3-kinase in caveolae to activate IkappaB kinase in hepatic macrophages. J Biol Chem 2007;282:5582–8. 10.1074/jbc.M609273200. 17172471

[65] Xiong S, She H, Takeuchi H, Han B, Engelhardt JF, Barton CH. et al. Signaling role of intracellular iron in NF-kappaB activation. J Biol Chem 2003;278:17646–54. 10.1074/jbc.M210905200. 12637578

[66] Sindrilaru A, Peters T, Wieschalka S, Baican C, Baican A, Peter H. et al. An unrestrained proinflammatory M1 macrophage population induced by iron impairs wound healing in humans and mice. J Clin Invest 2011;121:985–97. 10.1172/JCI44490. 21317534 PMC3049372

[67] Zhou Y, Que KT, Zhang Z, Yi ZJ, Zhao PX, You Y. et al. Iron overloaded polarizes macrophage to proinflammation phenotype through ROS/acetyl-p53 pathway. Cancer Med 2018;7:4012–22. 10.1002/cam4.1670. 29989329 PMC6089144

[68] Kroner A, Greenhalgh AD, Zarruk JG, Passos Dos Santos R, Gaestel M, David S. TNF and increased intracellular iron alter macrophage polarization to a detrimental M1 phenotype in the injured spinal cord. Neuron 2014;83:1098–116. 10.1016/j.neuron.2014.0. 25132469

[69] Mohammadpour M, Behjati M, Sadeghi A, Fassihi A. Wound healing by topical application of antioxidant iron chelators: kojic acid and deferiprone. Int Wound J 2013;10:260–4. 10.1111/j.1742-481X.2012.00971.x. 22621771 PMC7950824

[70] Guo J, Peng J, Han J, Wang K, Si R, Shan H. et al. Extracts of Portulaca oleracea promote wound healing by enhancing angiology regeneration and inhibiting iron accumulation in mice. Chin Herb Med 2022;14:263–72. 10.1016/j.chmed.2021.09.014. 36117668 PMC9476539

[71] Qin W, Liu K, Su H, Hou J, Yang S, Pan K. et al. Tibial cortex transverse transport promotes ischemic diabetic foot ulcer healing via enhanced angiogenesis and inflammation modulation in a novel rat model. Eur J Med Res 2024;29:155. 10.1186/s40001-024-01752-4. 38449025 PMC10918950

[72] Falanga V, Isseroff RR, Soulika AM, Romanelli M, Margolis D, Kapp S. et al. Chronic wounds. Nat Rev Dis Primers 2022;8:50. 10.1038/s41572-022-00377-3. 35864102 PMC10352385

[73] McDermott K, Fang M, Boulton AJM, Selvin E, Hicks CW. Etiology, epidemiology, and disparities in the burden of diabetic foot ulcers. Diabetes Care 2023;46:209–21. 10.2337/dci22-0043. 36548709 PMC9797649

[74] Harrison AV, Lorenzo FR, McClain DA. Iron and the pathophysiology of diabetes. Annu Rev Physiol 2023;85 (Volume 85, 2023):339–62. 10.1146/annurev-physiol-022522-102832. 36137277 PMC10161568

[75] Qasim N, Arif A, Mahmood R. Hyperglycemia enhances the generation of ROS and RNS that impair antioxidant power and cause oxidative damage in human erythrocytes. Biochem Cell Biol 2022;101:64–76. 10.1139/bcb-2022-0008. 36379031

[76] Volpe CMO, Villar-Delfino PH, dos Anjos PMF, Nogueira-Machado JA. Cellular death, reactive oxygen species (ROS) and diabetic complications. Cell Death Dis 2018;9:119. 10.1038/s41419-017-0135-z. 29371661 PMC5833737

[77] Duscher D, Neofytou E, Wong VW, Maan ZN, Rennert RC, Inayathullah M. et al. Transdermal deferoxamine prevents pressure-induced diabetic ulcers. Proc Natl Acad Sci 2015;112:94–9. 10.1073/pnas.1413445112. 25535360 PMC4291638

[78] Chen H, Jia P, Kang H, Zhang H, Liu Y, Yang P. et al. Upregulating Hif-1α by hydrogel Nanofibrous scaffolds for rapidly recruiting angiogenesis relative cells in diabetic wound. Adv Healthc Mater 2016;5:907–18. 10.1002/adhm.201501018. 26891197

[79] Cui S, Liu X, Liu Y, Hu W, Ma K, Huang Q. et al. Autophagosomes defeat Ferroptosis by decreasing generation and increasing discharge of free Fe2+ in skin repair cells to accelerate diabetic wound healing. Adv Sci 2023;10:e2300414. 10.1002/advs.202300414. PMC10477857 37387572

[80] Li S, Li Y, Wu Z, Wu Z, Fang H. Diabetic ferroptosis plays an important role in triggering on inflammation in diabetic wound. Am J Physiol Endocrinol Metab 2021;321:E509–20. 10.1152/ajpendo.00042.2021. 34423682

[81] Wang T, Zheng Y, Zhang J, Wu Z. Targeting ferroptosis promotes diabetic wound healing via Nrf2 activation. Heliyon 2024;10:e37477. 10.1016/j.heliyon.2024.e37477. 39421383 PMC11483302

[82] Yang X, Ren H, Guo X, Hu C, Fu J. Radiation-induced skin injury: pathogenesis, treatment, and management. Aging 2020;12:23379–93. 10.18632/aging.103932. 33202382 PMC7746368

[83] Ye LF, Chaudhary KR, Zandkarimi F, Harken AD, Kinslow CJ, Upadhyayula PS. et al. Radiation-induced lipid peroxidation triggers Ferroptosis and synergizes with Ferroptosis inducers. ACS Chem Biol 2020;15:469–84. 10.1021/acschembio.9b00939. 31899616 PMC7180072

[84] Lei G, Zhang Y, Koppula P, Liu X, Zhang J, Lin SH. et al. The role of ferroptosis in ionizing radiation-induced cell death and tumor suppression. Cell Res 2020;30:146–62. 10.1038/s41422-019-0263-3. 31949285 PMC7015061

[85] Li X, Zhuang X, Qiao T. Role of ferroptosis in the process of acute radiation-induced lung injury in mice. Biochem Biophys Res Commun 2019;519:240–5. 10.1016/j.bbrc.2019.08.165. 31493867

[86] Thermozier S, Hou W, Zhang X, Shields D, Fisher R, Bayir H. et al. Anti-Ferroptosis drug enhances Total-body irradiation mitigation by drugs that block apoptosis and necroptosis. Radiat Res 2020;193:435–50. 10.1667/RR15486.1. 32134361 PMC7299160

[87] Zhang X, Xing X, Liu H, Feng J, Tian M, Chang S. et al. Ionizing radiation induces ferroptosis in granulocyte-macrophage hematopoietic progenitor cells of murine bone marrow. Int J Radiat Biol 2020;96:584–95. 10.1080/09553002.2020.1708993. 31906761

[88] Vats K, Kruglov O, Mizes A, Samovich SN, Amoscato AA, Tyurin VA. et al. Keratinocyte death by ferroptosis initiates skin inflammation after UVB exposure. Redox Biol 2021;47:102143. 10.1016/j.redox.2021.102143. 34592565 PMC8487085

[89] Feng Z, Qin Y, Huo F, Jian Z, Li X, Geng J. et al. NMN recruits GSH to enhance GPX4-mediated ferroptosis defense in UV irradiation induced skin injury. Biochim Biophys Acta (BBA) - Mol Basis Dis 2022;1868:166287. 10.1016/j.bbadis.2021.166287. 34626772

[90] Nasir NJM, Heemskerk H, Jenkins J, Hamadee NH, Bunte R, Tucker-Kellogg L. Myoglobin-derived iron causes wound enlargement and impaired regeneration in pressure injuries of muscle. elife 2023;12:12. 10.7554/eLife.85633. PMC10238093 37267120

[91] Ishikawa M, Uchiyama A, Kosaka K, Nishio M, Ogino S, Yokoyama Y. et al. Exposure to volatile ferroptosis inhibitor, TEMPO, reduced cutaneous ischemia-reperfusion injury progression to pressure ulcer formation in a mouse model. J Dermatol Sci 2024;115:130–40. 10.1016/j.jdermsci.2024.07.005. 39098373

[92] Caggiati A, Rosi C, Casini A, Cirenza M, Petrozza V, Acconcia MC. et al. Skin iron deposition characterises lipodermatosclerosis and leg ulcer. Eur J Vasc Endovasc Surg 2010;40:777–82. 10.1016/j.ejvs.2010.08.015. 20880725

[93] Wenk J, Foitzik A, Achterberg V, Sabiwalsky A, Dissemond J, Meewes C. et al. Selective pick-up of increased iron by deferoxamine-coupled cellulose abrogates the iron-driven induction of matrix-degrading metalloproteinase 1 and lipid peroxidation in human dermal fibroblasts in vitro: a new dressing concept. J Invest Dermatol 2001;116:833–9. 10.1046/j.1523-1747.2001.01345.x. 11407968

[94] Yeoh-Ellerton S, Stacey MC. Iron and 8-isoprostane levels in acute and chronic wounds. J Invest Dermatol 2003;121:918–25. 10.1046/j.1523-1747.2003.12471.x. 14632213

[95] Yao X, Zhang Y, Hao J, Duan HQ, Zhao CX, Sun C. et al. Deferoxamine promotes recovery of traumatic spinal cord injury by inhibiting ferroptosis. Neural Regen Res 2019;14:532–41. 10.4103/1673-5374.245480. 30539824 PMC6334606

[96] Bao Z, Liu Y, Chen B, Miao Z, Tu Y, Li C. et al. Prokineticin-2 prevents neuronal cell deaths in a model of traumatic brain injury. Nat Commun 2021;12:4220. 10.1038/s41467-021-24469-y. 34244497 PMC8270965

[97] Li QS, Jia YJ. Ferroptosis: a critical player and potential therapeutic target in traumatic brain injury and spinal cord injury. Neural Regen Res 2023;18:506–12. 10.4103/1673-5374.350187. 36018155 PMC9727428

[98] Fang J, Yuan Q, Du Z, Zhang Q, Yang L, Wang M. et al. Overexpression of GPX4 attenuates cognitive dysfunction through inhibiting hippocampus ferroptosis and neuroinflammation after traumatic brain injury. Free Radic Biol Med 2023;204:68–81. 10.1016/j.freeradbiomed.2023.04.014. 37105419

[99] Xie B-S, Wang Y-Q, Lin Y, Mao Q, Feng J-F, Gao G-Y. et al. Inhibition of ferroptosis attenuates tissue damage and improves long-term outcomes after traumatic brain injury in mice. CNS Neurosci Ther 2019;25:465–75. 10.1111/cns.13069. 30264934 PMC6488926

[100] Pastar I, Stojadinovic O, Yin NC, Ramirez H, Nusbaum AG, Sawaya A. et al. Epithelialization in wound healing: a comprehensive review. Adv Wound Care 2014;3:445–64. 10.1089/wound.2013.0473. PMC4086220 25032064

[101] Al , Sadoun H. Macrophage phenotypes in normal and diabetic wound healing and therapeutic interventions. Cells 2022;11:2430. 10.3390/cells11152430. PMC9367932 35954275

[102] Cheng H, Huang H, Guo Z, Chang Y, Li Z. Role of prostaglandin E2 in tissue repair and regeneration. Theranostics 2021;11:8836–54. 10.7150/thno.63396. 34522214 PMC8419039

[103] Zhang S, Liu Y, Zhang X, Zhu D, Qi X, Cao X. et al. Prostaglandin E(2) hydrogel improves cutaneous wound healing via M2 macrophages polarization. Theranostics 2018;8:5348–61. 10.7150/thno.27385. 30555551 PMC6276096

[104] Xu Y, Liu Y, Li K, Yuan D, Yang S, Zhou L. et al. COX-2/PGE2 pathway inhibits the Ferroptosis induced by cerebral ischemia reperfusion. Mol Neurobiol 2022;59:1619–31. 10.1007/s12035-021-02706-1. 35013936

[105] Wang B, Jin Y, Liu J, Liu Q, Shen Y, Zuo S. et al. EP1 activation inhibits doxorubicin-cardiomyocyte ferroptosis via Nrf2. Redox Biol 2023;65:102825. 10.1016/j.redox.2023.102825. 37531930 PMC10400469

[106] DiPietro LA . Angiogenesis and wound repair: when enough is enough. J Leukoc Biol 2016;100:979–84. 10.1189/jlb.4MR0316-102R. 27406995 PMC6608066

[107] Cao G, Yin S, Ma J, Lu Y, Song R, Wu Z. et al. YAP promotes the healing of ischemic wounds by reducing ferroptosis in skin fibroblasts through inhibition of ferritinophagy. Heliyon 2024;10:e24602. 10.1016/j.heliyon.2024.e24602. 38298641 PMC10828694

[108] Luo EF, Li HX, Qin YH, Qiao Y, Yan GL, Yao YY. et al. Role of ferroptosis in the process of diabetes-induced endothelial dysfunction. World J Diabetes 2021;12:124–37. 10.4239/wjd.v12.i2.124. 33594332 PMC7839168

[109] Pasaribu KM, Ilyas S, Tamrin T, Radecka I, Swingler S, Gupta A. et al. Bioactive bacterial cellulose wound dressings for burns with collagen in-situ and chitosan ex-situ impregnation. Int J Biol Macromol 2023;230:123118. 10.1016/j.ijbiomac.2022.123118. 36599383

[110] Broughton G, 2nd, Janis JE, Attinger CE. Wound healing: an overview. Plast Reconstr Surg 2006;117:1e–32e-S. 10.1097/01.prs.0000222562.60260.f9. 16801750

[111] Velnar T, Bailey T, Smrkolj V. The wound healing process: an overview of the cellular and molecular mechanisms. J Int Med Res 2009;37:1528–42. 10.1177/147323000903700531. 19930861

[112] Gonzalez AC, Costa TF, Andrade ZA, Medrado AR. Wound healing - a literature review. An Bras Dermatol 2016;91:614–20. 10.1590/abd1806-4841.20164741. 27828635 PMC5087220

[113] Ezzo M, Hinz B. Novel approaches to target fibroblast mechanotransduction in fibroproliferative diseases. Pharmacol Ther 2023;250:108528. 10.1016/j.pharmthera.2023.108528. 37708995

[114] Schuster R, Younesi F, Ezzo M, Hinz B. The role of Myofibroblasts in physiological and pathological tissue repair. Cold Spring Harb Perspect Biol 2023;15:a041231. 10.1101/cshperspect.a041231. PMC9808581 36123034

[115] Tian H, Li S, Jia W, Yu K, Wu G. Risk factors for poor hemostasis of prophylactic uterine artery embolization before curettage in cesarean scar pregnancy. J Int Med Res 2020;48:300060520964379. 10.1177/0300060520964379. 33467974 PMC7967858

[116] Yuan T, Meijia L, Rong C, Jian Y, Lijun H. Identification of novel biomarkers of ferroptosis involved in keloid based on bioinformatics analysis. Int Wound J 2024;21:e14606. 10.1111/iwj.14606. 38272797 PMC10805535

[117] Zhang J, Liu L, Li X, Shen X, Yang G, Deng Y. et al. 5-ALA-PDT induced ferroptosis in keloid fibroblasts via ROS, accompanied by downregulation of xCT, GPX4. Photodiagn Photodyn Ther 2023;42:103612. 10.1016/j.pdpdt.2023.103612. 37220842

[118] Yang L, Li X, Wang Y. Ferrostatin-1 inhibits fibroblast fibrosis in keloid by inhibiting ferroptosis. PeerJ 2024;12:e17551. 10.7717/peerj.17551. 38887622 PMC11182022

[119] Recalcati S, Gammella E, Buratti P, Doni A, Anselmo A, Locati M. et al. Macrophage ferroportin is essential for stromal cell proliferation in wound healing. Haematologica 2019;104:47–58. 10.3324/haematol.2018.197517. 30115660 PMC6312033

[120] Wang H, An P, Xie EJ, Wu Q, Fang XX, Gao H. et al. Characterization of ferroptosis in murine models of hemochromatosis. Hepatology 2017;66:449–65. 10.1002/hep.29117. 28195347 PMC5573904

[121] Skouta R, Dixon SJ, Wang J, Dunn DE, Orman M, Shimada K. et al. Ferrostatins inhibit oxidative lipid damage and cell death in diverse disease models. J Am Chem Soc 2014;136:4551–6. 10.1021/ja411006a. 24592866 PMC3985476

[122] Hofmans S, Berghe TV, Devisscher L, Hassannia B, Lyssens S, Joossens J. et al. Novel Ferroptosis inhibitors with improved potency and ADME properties. J Med Chem 2016;59:2041–53. 10.1021/acs.jmedchem.5b01641. 26696014

[123] Mizuno H, Kubota C, Takigawa Y, Shintoku R, Kannari N, Muraoka T. et al. 2,2,6,6-Tetramethylpiperidine-1-oxyl acts as a volatile inhibitor of ferroptosis and neurological injury. J Biochem 2022;172:71–8. 10.1093/jb/mvac044. 35512114

[124] Zilka O, Poon J-F, Pratt DA. Radical-trapping antioxidant activity of copper and nickel Bis(Thiosemicarbazone) complexes underlies their potency as inhibitors of Ferroptotic cell death. J Am Chem Soc 2021;143:19043–57. 10.1021/jacs.1c08254. 34730342

[125] Soula M, Weber RA, Zilka O, Alwaseem H, La K, Yen F. et al. Metabolic determinants of cancer cell sensitivity to canonical ferroptosis inducers. Nat Chem Biol 2020;16:1351–60. 10.1038/s41589-020-0613-y. 32778843 PMC8299533

[126] Duan W, Ou Z, Huang Y, Zhang Y, Zhang L, Zhao Y. et al. Silibinin inhibits cell Ferroptosis and Ferroptosis-related tissue injuries. Antioxidants (Basel, Switzerland) 2023;12:2119. 10.3390/antiox12122119. PMC10740598 38136238

[127] Wang W, Zhai T, Luo P, Miao X, Wang J, Chen Y. Beneficial effects of silibinin on serum lipids, bile acids, and gut microbiota in methionine-choline-deficient diet-induced mice. Front Nutr 2023;10:1257158. 10.3389/fnut.2023.1257158. 37867498 PMC10587424

[128] Wortmann M, Schneider M, Pircher J, Hellfritsch J, Aichler M, Vegi N. et al. Combined deficiency in glutathione peroxidase 4 and vitamin E causes multiorgan thrombus formation and early death in mice. Circ Res 2013;113:408–17. 10.1161/CIRCRESAHA.113.279984. 23770613

[129] Carlson BA, Tobe R, Yefremova E, Tsuji PA, Hoffmann VJ, Schweizer U. et al. Glutathione peroxidase 4 and vitamin E cooperatively prevent hepatocellular degeneration. Redox Biol 2016;9:22–31. 10.1016/j.redox.2016.05.003. 27262435 PMC4900515

[130] Altamura S, Vegi NM, Hoppe PS, Schroeder T, Aichler M, Walch A. et al. Glutathione peroxidase 4 and vitamin E control reticulocyte maturation, stress erythropoiesis and iron homeostasis. Haematologica 2020;105:937–50. 10.3324/haematol.2018.212977. 31248967 PMC7109755

[131] Hu Q, Zhang Y, Lou H, Ou Z, Liu J, Duan W. et al. GPX4 and vitamin E cooperatively protect hematopoietic stem and progenitor cells from lipid peroxidation and ferroptosis. Cell Death Dis 2021;12:706. 10.1038/s41419-021-04008-9. 34267193 PMC8282880

[132] Kontoghiorghes GJ . Ethical issues and risk/benefit assessment of iron chelation therapy: advances with deferiprone/deferoxamine combinations and concerns about the safety, efficacy and costs of deferasirox. Hemoglobin 2008;32:1–15. 10.1080/03630260701726533. 18274978

[133] Zhu H, Cen J, Hong C, Wang H, Wen Y, He Q. et al. Targeting labile iron-mediated Ferroptosis provides a potential therapeutic strategy for rhabdomyolysis-induced acute kidney injury. ACS Chem Biol 2023;18:1294–304. 10.1021/acschembio.2c00914. 37172039

[134] Wang C, Xie L, Xing Y, Liu M, Yang J, Gao N. et al. Iron-overload-induced ferroptosis in mouse cerebral toxoplasmosis promotes brain injury and could be inhibited by Deferiprone. PLoS Negl Trop Dis 2023;17:e0011607. 10.1371/journal.pntd.0011607. 37651502 PMC10508604

[135] Hider RC, Hoffbrand AV. The role of Deferiprone in iron chelation. N Engl J Med 2018;379:2140–50. 10.1056/NEJMra1800219. 30485781

[136] Alim I, Caulfield JT, Chen Y, Swarup V, Geschwind DH, Ivanova E. et al. Selenium drives a transcriptional adaptive program to block Ferroptosis and treat stroke. Cell 2019;177:1262–1279.e25. 10.1016/j.cell.2019.03.032. 31056284

[137] Shchepinov MS . Polyunsaturated fatty acid Deuteration against neurodegeneration. Trends Pharmacol Sci 2020;41:236–48. 10.1016/j.tips.2020.01.010. 32113652

[138] Zesiewicz T, Heerinckx F, De Jager R, Omidvar O, Kilpatrick M, Shaw J. et al. Randomized, clinical trial of RT001: early signals of efficacy in Friedreich's ataxia. Mov Disord 2018;33:1000–5. 10.1002/mds.27353. 29624723

